# Global, regional, and national burden of NAFLD in youths and young adults aged 15–39 years, 1990–2021, its attributable risk factors, and projections to 2035: a systematic analysis of the Global Burden of Disease Study 2021

**DOI:** 10.3389/fnut.2025.1509232

**Published:** 2025-01-28

**Authors:** Jiong Wang, Jiqing Du, Minxiu Wang, Mengyun Jin, Zhihua Tang, Yuqin Mao

**Affiliations:** ^1^Department of Pharmacy, Shaoxing People's Hospital, Shaoxing, China; ^2^School of Life and Health Technology, Dongguan University of Technology, Dongguan, China

**Keywords:** non-alcoholic fatty liver disease, Global Burden of Disease, young adults, youths, risk factors

## Abstract

Non-alcoholic fatty liver disease (NAFLD) is a significant health burden in youths and young adults, and the trend toward younger onset of NAFLD is alarming. Utilizing data from the Global Burden of Disease (GBD) 2021 study, this study assessed the burden and trends of NAFLD in youths and young adults aged 15–39 from 1990 to 2021 and extracted data from this study on the incidence, prevalence, death, and disability-adjusted life-years (DALYs) rates of NAFLD. We evaluated the global temporal trend of NAFLD from 1990 to 2021 with estimated annual percentage change (EAPC) and age-standardized rate (ASR). The Bayesian age-period-cohort (BAPC) model was used to predict future trends of the NAFLD burden to 2035. We found that the global burden of NAFLD in youths and young adults has risen steadily from 1990 to 2021, and projects to increase to 2035, which places enormous pressure on society. To alleviate this burden, implementing measures targeting risk factors such as glycemic control and smoking cessation is necessary.

## Introduction

Non-alcoholic fatty liver disease (NAFLD) is a chronic liver disease with high global prevalence, which has emerged as the fastest-growing contributor to liver morbidity and mortality (1, 2). Based on epidemiological evidence, the global prevalence of NAFLD increased from 25.3% in 1990–2006 to 38.0% in 2016–2019 (3). NAFLD exhibits a high overall global prevalence that affects individuals across all age groups and is particularly prevalent in younger populations in recent years, estimated at a worldwide prevalence of 7.4% (4, 5). This phenomenon has considerably diminished their quality of life (5, 6). The incidence of obesity and type 2 diabetes mellitus (T2DM) is also increasing among young people due to unhealthy diets and reduced physical activity, portending a tremendous burden of NAFLD on the socio-medical and health systems (7).

Studies have shown that more than 10% of NAFLD patients may develop nonalcoholic steatohepatitis (NASH), with steatosis, hepatocyte ballooning, lobular inflammation, and fibrosis as its hallmarks (8, 9). In the presence of tissue damage, it may progress to end-stage cirrhosis and liver cancer (10). In addition, despite advances in research on NAFLD, effective pharmacological treatment strategies are still lacking (11, 12).

While existing studies have explored the global impact of NAFLD, there is a substantial gap in studies targeting the 15–39 age group (13). Therefore, considering the crucial role of adolescents in society, we aimed to delineate the global burden of NAFLD among adolescents and young adults aged 15–39 years from 1990 to 2021 with data from the Global Burden of Disease (GBD) 2021 study. There was a rise in age-standardized prevalence or incidence of NAFLD in all 21 regions studied over 30 years and projecting to the next decade.

## Materials and methods

### Study data

This study utilized data on the incidence, prevalence, deaths, and DALYs attributable to NAFLD and the relevant risk factors from 1990 to 2021 acquired from the Global Health Data Exchange (GHDx) query tool,^1^ based on gender, age, region, and country. This study categorized 204 countries and territories into five levels based on their Socio-Demographic Index (SDI), which includes high, high-middle, middle, low-middle, and low SDI. We calculated the SDI through several social factors, with the fertility rate of the population aged <25 years, the education level of the population aged >15 years, and per capita income (14). According to geographic continuity, the world was divided geographically into 21 distinct GBD regions, such as Western Europe and East Asia. Employing the latest epidemiological data and refined standardized methods, the GBD 2021 study offers a comprehensive evaluation of health loss linked to 371 diseases, injuries, and impairments, as well as 87 risk factors in 204 nations and territories (15, 16). The NAFLD data derived from the GHDx Query Tool did not require patient consent as it was publicly available and anonymized. These entries did not include individual patient-level data but were aggregated in a summary format at the regional or national level.

In this study, we gathered data on NAFLD in 21 national regions. These areas had similar geographic locations and epidemiologic characteristics covering men and women in five age groups (15–19, 20–24, 25–29, 30–34, and 35–39 years).

### Statistical analysis

The trends for NAFLD incidence, prevalence, and mortality rates were evaluated by calculating the age-standardized incidence rate (ASIR), age-standardized prevalence rate (ASPR), age-standardized mortality rate (ASMR), age-standardized DALYs rate (ASDR), and their respective estimated annual percentage change (EAPC). DALYs were computed as the sum of the years lived with disability and the years of life lost. According to the age group construction of the standard population, the ASR (per100, 000 population) were calculated using the following formula:


$$ASR=\frac{\sum_{i=1}^{A}a_{i}w_{i}}{\sum_{i=1}^{A}a_{i}}\times 100,000\text{,}$$

*a_i_* refers to the incidence of the *i*th age group. *w_i_* denotes the number of persons (or weight) in the same age subgroup *i* of the assigned reference standard population.

EAPC is used to estimate the trends of the ASRs, and it quantitatively calculates the average annual rate of change of ASR for a specified period (17), which follows this formula, i.e., y=α+βx+ε , where *y* = ln (ASR), and *x* = the calendar year. The EAPC calculation formula, 100 × (exp(*β*) − 1), and its 95% confidence intervals (CI) can also be calculated from the linear regression model.

Estimation of GBD risk factors is based on a comparative risk assessment framework and involves six steps. (1) identifying the inclusion of risk-outcome pairs; (2) estimation of relative risk as functional exposure; (3) estimation of exposures for each risk by age, sex, location, and year; (4) identification of the theoretical minimum risk exposure level (TMREL) and the counterfactual exposure; (5) estimation of attributable burden and population attributable fractions (PAFs); and (6) estimation of the deaths and DALYs attributable to various combinations of risk factors.

The Bayesian age-period-cohort (BAPC) model can achieve more reasonable predictions to the global burden trends. Based on the assumption that the effects of age, period, and cohort were similar in temporal proximity, Bayesian inference in the BAPC model utilized a second-order stochastic excursion to smooth the prior three aforementioned values and forecast the posterior rates. An integrated nested Laplacian approximation was used with this BAPC model to approximate marginal posterior distributions, avoiding some of the mixing and convergence problems introduced by the Markov Chain Monte Carlo sampling technique traditionally used for Bayesian methods (18).

All data analyses were performed using the open-source software R (version 4.3.3). Statistical significance was set at *p* < 0.05.

## Results

### NAFLD burden at global level

Between 1990 and 2021, the global incident cases of NAFLD increased by 71%, from 17.05 million (95% UI: 14.70.58–19.77) to 29.08 million (95% UI: 24.99–33.49). The age-standardized incidence rate (ASIR) of NAFLD among the 19–35 age group increased by 26%, from 778.04 per 100,000 population (95% UI: 670.53–901.83) in 1990 to 977.61 per 100,000 population (95% UI: 839.97–1125.92) in 2021, with an estimated annual percentage change (EAPC) of 0.79 (Table 1).

**Table 1:** Incident cases and ASIR of NAFLD in 1990 and 2021, and temporal trends.

	1990		2021		1990–2021 EAPC of ASIR (95%CI)
	Incident cases, No. × 10^3^	ASIR per 100,000 (95% UI)	Incident cases, No. × 10^3^	ASIR per 100,000 (95% UI)	
Global	17053.03 (14696.58–19766.30)	778.04 (670.53–901.83)	29082.23 (24987.55–33494.07)	977.61 (839.97–1125.92)	0.79 (0.74–0.84)
Socio-demographic index					
Low SDI	1390.35 (1198.33–1622.70)	754.36 (650.18–880.43)	4007.25 (3468.37–4633.08)	892.36 (772.36–1031.72)	0.54 (0.50–0.57)
Low-middle SDI	3702.34 (3201.37–4299.77)	816.57 (706.08–948.34)	8106.88 (7005.82–9380.10)	1010.21 (873.01–1168.87)	0.70 (0.66–0.73)
Middle SDI	6458.00 (5584.65–7483.46)	858.06 (742.02–994.30)	9958.16 (8548.22–11475.57)	1073.67 (921.65–1237.27)	0.79 (0.73–0.84)
High-middle SDI	3562.16 (3063.90–4148.05)	787.15 (677.05–916.62)	4387.55 (3764.79–5076.17)	996.56 (855.12–1152.98)	0.83 (0.70–0.95)
High SDI	1924.09 (1658.87–2236.00)	554.55 (478.11–644.45)	2599.28 (2242.59–2996.57)	735.84 (634.86–848.31)	1.12 (1.05–1.20)
Region					
Andean Latin America	128.48 (111.96–148.49)	830.82 (724.03–960.23)	272.28 (236.96–312.59)	1005.50 (875.05–1154.33)	0.65 (0.64–0.66)
Australasia	38.38 (33.35–44.18)	470.65 (408.96–541.79)	61.68 (53.62–71.09)	589.11 (512.04–678.95)	0.81 (0.75–0.86)
Caribbean	123.59 (107.39–143.81)	831.45 (722.46–967.44)	172.14 (148.88–197.99)	945.69 (817.92–1087.68)	0.53 (0.49–0.57)
Central Asia	252.47 (217.75–291.90)	887.30 (765.29–1025.86)	384.23 (330.62–442.62)	1027.72 (884.32–1183.89)	0.61 (0.56–0.65)
Central Europe	324.66 (281.94–375.30)	693.01 (601.81–801.09)	273.55 (236.94–315.57)	781.12 (676.60–901.13)	0.34 (0.32–0.36)
Central Latin America	673.95 (582.15–779.84)	987.22 (852.74–1142.34)	1171.60 (1011.09–1346.92)	1158.14 (999.47–1331.44)	0.56 (0.54–0.57)
Central Sub-Saharan Africa	146.03 (124.83–170.86)	703.36 (601.25–822.93)	415.52 (357.92–482.02)	768.12 (661.63–891.05)	0.26 (0.21–0.30)
East Asia	4409.71 (3781.47–5134.16)	779.51 (668.45–907.57)	4682.78 (3997.14–5482.31)	977.52 (834.39–1144.42)	0.85 (0.60–1.10)
Eastern Europe	566.23 (486.13–653.55)	660.19 (566.79–761.99)	491.64 (422.04–571.95)	742.95 (637.77–864.32)	0.39 (0.35–0.43)
Eastern Sub-Saharan Africa	535.14 (462.17–623.00)	754.89 (651.96–878.83)	1509.24 (1302.27–1751.92)	861.51 (743.36–1000.04)	0.43 (0.40–0.46)
High-income Asia Pacific	349.89 (304.37–405.59)	518.40 (450.95–600.92)	274.93 (237.02–317.36)	543.98 (468.98–627.94)	0.36 (0.25–0.47)
High-income North America	542.40 (465.93–633.93)	478.66 (411.18–559.44)	731.38 (632.16–851.33)	593.73 (513.18–691.10)	0.83 (0.79–0.87)
North Africa and Middle East	2045.90 (1779.87–2357.47)	1528.74 (1329.95–1761.54)	4669.45 (4036.93–5318.39)	1836.44 (1587.68–2091.66)	0.63 (0.57–0.68)
Oceania	23.39 (20.06–27.14)	880.64 (755.17–1021.74)	54.14 (46.67–62.49)	960.86 (828.24–1109.16)	0.28 (0.24–0.33)
South Asia	2944.98 (2529.95–3438.67)	682.32 (586.16–796.70)	6856.98 (5876.62–7993.70)	866.95 (743.00–1010.67)	0.76 (0.68–0.83)
Southeast Asia	1692.61 (1456.50–1974.65)	859.17 (739.32–1002.34)	2785.60 (2386.59–3217.59)	1004.45 (860.57–1160.22)	0.55 (0.53–0.58)
Southern Latin America	93.99 (80.63–109.23)	492.61 (422.62–572.49)	158.58 (136.19–185.00)	614.75 (527.95–717.16)	0.73 (0.68–0.78)
Southern Sub-Saharan Africa	207.86 (178.36–240.66)	961.62 (825.17–1113.39)	381.35 (327.84–438.26)	1120.43 (963.21–1287.63)	0.57 (0.54–0.60)
Tropical Latin America	574.94 (500.10–667.76)	893.98 (777.60–1038.30)	942.63 (813.39–1091.36)	1067.41 (921.06–1235.83)	0.66 (0.62–0.69)
Western Europe	766.72 (665.54–884.96)	532.00 (461.80–614.05)	857.66 (744.44–984.85)	660.89 (573.65–758.90)	0.80 (0.75–0.85)
Western Sub-Saharan Africa	611.25 (526.59–707.64)	854.01 (735.73–988.68)	1934.70 (1668.21–2238.22)	1011.83 (872.46–1170.56)	0.55 (0.54–0.56)

ASIR, Age-standardized incidence rate; EAPC, Estimated annual percentage change; CI, Confidence interval; SDI, Socio-demographic index; UI, Uncertainty interval.

Similarly, the prevalent cases of NAFLD increased by 76% between 1990 and 2021, from 0.24 billion (95% UI: 0.21–0.28) to 0.42 billion (95% UI: 0.37–0.49). The age-standardized prevalence rate (ASPR) of NAFLD among the 19–35 age group showed an increase of 29%, from 11010.48 per 100,000 population (95% UI: 9515.14–12829.00) in 1990 to 14221.32 per 100,000 population (95% UI: 12322.00–16615.70) in 2021, with an EAPC of 0.84 (Table 2).

**Table 2:** Prevalent cases and ASPR of NAFLD in 1990 and 2021, and temporal trends.

	1990		2021		1990–2021 EAPC of ASPR (95%CI)
	Prevalent cases, No. × 10^3^	ASPR per 100,000 (95% UI)	Prevalent cases, No. × 10^3^	ASPR per 100,000 (95% UI)	
Global	241327.58 (208552.73–281185.67)	11010.48 (9515.14–12829.00)	423059.63 (366558.18–494288.31)	14221.32 (12322.00–16615.70)	0.84 (0.78–0.91)
Socio-demographic index					
Low SDI	19403.70 (16727.85–22425.51)	10527.84 (9076.01–12167.37)	53404.64 (46029.74–61815.79)	11892.49 (10250.20–13765.54)	0.40 (0.37–0.43)
Low-middle SDI	52736.71 (45290.19–61262.34)	11631.38 (9989.01–13511.76)	113704.98 (98430.82–131889.05)	14168.98 (12265.64–16434.92)	0.63 (0.57–0.69)
Middle SDI	88203.41 (76176.91–102367.44)	11719.33 (10121.40–13601.26)	143904.88 (124277.07–167740.25)	15515.52 (13399.29–18085.40)	0.91 (0.84–0.98)
High-middle SDI	51058.71 (44111.92–59662.28)	11282.76 (9747.68–13183.94)	68267.04 (58803.74–79934.87)	15505.82 (13356.38–18155.99)	1.04 (0.89–1.20)
High SDI	29707.39 (25652.82–34617.17)	8562.08 (7393.50–9977.15)	43452.54 (37464.11–50742.29)	12301.11 (10605.83–14364.79)	1.32 (1.26–1.38)
Region					
Andean Latin America	1627.32 (1402.98–1897.53)	10523.49 (9072.74–12270.89)	3557.02 (3075.00–4139.83)	13135.46 (11355.45–15287.68)	0.77 (0.76–0.79)
Australasia	588.88 (502.93–684.56)	7222.13 (6167.95–8395.51)	998.13 (855.44–1160.08)	9532.41 (8169.70–11079.03)	0.86 (0.83–0.88)
Caribbean	1691.21 (1462.18–1957.40)	11377.42 (9836.62–13168.16)	2430.07 (2097.58–2835.04)	13350.04 (11523.45–15574.82)	0.45 (0.41–0.48)
Central Asia	3574.12 (3073.49–4169.81)	12561.16 (10801.70–14654.70)	5779.62 (4978.14–6769.17)	15458.97 (13315.22–18105.77)	0.62 (0.49–0.75)
Central Europe	4980.32 (4290.39–5816.71)	10630.71 (9158.03–12416.02)	4426.41 (3802.00–5153.87)	12639.77 (10856.75–14717.03)	0.71 (0.63–0.79)
Central Latin America	8306.98 (7210.34–9682.66)	12168.33 (10561.93–14183.46)	15308.62 (13196.41–17836.47)	15132.69 (13044.75–17631.49)	0.70 (0.69–0.72)
Central Sub-Saharan Africa	1942.69 (1668.74–2271.68)	9356.86 (8037.41–10941.43)	5560.80 (4766.15–6472.60)	10279.49 (8810.53–11965.01)	0.30 (0.26–0.33)
East Asia	61946.60 (53314.35–72153.04)	10950.31 (9424.39–12754.51)	72557.36 (62072.29–85611.92)	15146.16 (12957.42–17871.26)	1.01 (0.75–1.27)
Eastern Europe	8600.78 (7403.94–10068.45)	10027.90 (8632.47–11739.10)	7806.46 (6672.72–9175.87)	11796.99 (10083.71–13866.42)	0.57 (0.43–0.71)
Eastern Sub-Saharan Africa	7049.75 (6083.45–8145.49)	9944.66 (8581.56–11490.35)	19679.18 (16945.13–22846.71)	11233.34 (9672.69–13041.45)	0.42 (0.38–0.45)
High-income Asia Pacific	5146.06 (4472.08–5945.19)	7624.32 (6625.78–8808.32)	4649.60 (3989.45–5422.29)	9199.95 (7893.75–10728.84)	0.74 (0.66–0.81)
High-income North America	8631.58 (7400.27–10043.56)	7617.30 (6530.68–8863.35)	11923.01 (10274.47–13858.28)	9679.01 (8340.74–11250.05)	0.83 (0.78–0.88)
North Africa and Middle East	26261.74 (22816.88–30421.86)	19623.28 (17049.22–22731.81)	69336.38 (60150.17–80157.80)	27269.21 (23656.38–31525.15)	1.20 (1.14–1.26)
Oceania	283.54 (246.41–329.17)	10673.92 (9276.30–12391.81)	678.04 (584.07–792.14)	12033.84 (10366.16–14059.03)	0.39 (0.35–0.42)
South Asia	45788.03 (39355.68–53201.27)	10608.50 (9118.21–12326.05)	100017.48 (86161.77–116387.96)	12645.57 (10893.74–14715.34)	0.53 (0.43–0.63)
Southeast Asia	22965.30 (19749.66–26610.31)	11657.24 (10024.97–13507.45)	38433.73 (32997.71–44970.72)	13858.66 (11898.51–16215.81)	0.57 (0.56–0.59)
Southern Latin America	1407.20 (1201.95–1642.26)	7375.55 (6299.80–8607.56)	2519.41 (2151.71–2943.56)	9766.77 (8341.36–11411.03)	0.91 (0.89–0.94)
Southern Sub-Saharan Africa	2572.95 (2232.96–2995.79)	11903.36 (10330.44–13859.58)	5060.28 (4347.22–5924.50)	14867.43 (12772.41–17406.57)	0.75 (0.68–0.82)
Tropical Latin America	8066.77 (6925.00–9417.95)	12542.97 (10767.64–14643.91)	13401.12 (11558.00–15689.12)	15175.15 (13088.04–17766.03)	0.69 (0.64–0.73)
Western Europe	11564.43 (9964.46–13449.73)	8024.16 (6914.00–9332.31)	14092.04 (12168.54–16463.86)	10859.02 (9376.81–12686.69)	0.98 (0.91–1.06)
Western Sub-Saharan Africa	8331.34 (7171.09–9652.40)	11640.17 (10019.13–13485.90)	24844.87 (21497.05–28874.53)	12993.60 (11242.72–15101.07)	0.35 (0.34–0.36)

ASIR, Age-standardized prevalence rate; EAPC, Estimated annual percentage change; CI, Confidence interval; SDI, Socio-demographic index; UI, Uncertainty interval.

The fatal cases and DALYs of NAFLD also grew between 1990 and 2021. Fatal cases increased from 3.82 thousand (95% UI: 2.56–5.60) to 6.45 thousand (95% UI: 4.20–9.42), and the number of DALYs rose from 225.36 thousand (95% UI: 153.05–328.28) to 377.55 thousand (95% UI: 246.02–548.56). The age-standardized mortality rate (ASMR) and age-standardized DALYs rate (ASDR) also saw a rise. ASMR increased from 0.17 per 100,000 population (95% UI: 0.12–0.26) in 1990 to 0.22 per 100,000 population (95% UI: 0.14–0.32) in 2021 (EAPC = 0.67). ASDR went from 10.28 per 100,000 population (95% UI: 6.98–14.98) in 1990 to 12.69 per 100,000 population (95% UI: 8.27–18.44) in 2021 (EAPC = 0.65) (Tables 3, 4).

**Table 3:** Fatal cases and ASMR of NAFLD in 1990 and 2021, and temporal trends.

	1990		2021		1990–2021 EAPC of ASMR (95%CI)
	Fatal cases, No. × 10^3^	ASMR per 100,000 (95% UI)	Fatal cases, No. × 10^3^	ASMR per 100,000 (95% UI)	
Global	3.82 (2.56–5.60)	0.17 (0.12–0.26)	6.45 (4.20–9.42)	0.22 (0.14–0.32)	0.67 (0.57–0.77)
Socio-demographic index					
Low SDI	0.32 (0.22–0.47)	0.17 (0.12–0.25)	0.78 (0.51–1.11)	0.17 (0.11–0.25)	−0.03(−0.11–0.04)
Low-middle SDI	0.82 (0.54–1.21)	0.18 (0.12–0.27)	1.71 (1.11–2.51)	0.21 (0.14–0.31)	0.54 (0.43–0.65)
Middle SDI	1.45 (0.99–2.09)	0.19 (0.13–0.28)	2.22 (1.44–3.27)	0.24 (0.16–0.35)	0.53 (0.43–0.62)
High-middle SDI	0.65 (0.44–0.94)	0.14 (0.10–0.21)	1.16 (0.71–1.79)	0.26 (0.16–0.41)	2.20 (1.75–2.66)
High SDI	0.58 (0.35–0.89)	0.17 (0.10–0.26)	0.58 (0.37–0.87)	0.16 (0.10–0.25)	−0.34(−0.60--0.08)
Region					
Andean Latin America	0.08 (0.05–0.12)	0.51 (0.30–0.79)	0.12 (0.07–0.19)	0.45 (0.27–0.70)	−0.63(−0.81--0.44)
Australasia	0.01 (0.01–0.01)	0.10 (0.06–0.15)	0.01 (0.01–0.02)	0.12 (0.09–0.18)	0.63 (0.48–0.78)
Caribbean	0.05 (0.03–0.08)	0.32 (0.19–0.51)	0.07 (0.04–0.11)	0.36 (0.20–0.58)	0.40 (0.05–0.75)
Central Asia	0.08 (0.05–0.12)	0.28 (0.17–0.43)	0.24 (0.14–0.38)	0.65 (0.37–1.03)	2.31 (1.90–2.73)
Central Europe	0.09 (0.05–0.15)	0.20 (0.11–0.31)	0.08 (0.05–0.13)	0.23 (0.13–0.37)	0.09(−0.40–0.57)
Central Latin America	0.31 (0.18–0.49)	0.46 (0.26–0.72)	0.64 (0.38–1.01)	0.63 (0.37–1.00)	0.88 (0.65–1.11)
Central Sub-Saharan Africa	0.04 (0.02–0.07)	0.20 (0.11–0.33)	0.11 (0.06–0.17)	0.20 (0.11–0.31)	−0.10(−0.24–0.04)
East Asia	0.64 (0.47–0.85)	0.11 (0.08–0.15)	0.44 (0.31–0.61)	0.09 (0.06–0.13)	−1.25(−1.60--0.90)
Eastern Europe	0.11 (0.07–0.18)	0.13 (0.08–0.21)	0.72 (0.38–1.17)	1.08 (0.57–1.77)	6.96 (5.69–8.25)
Eastern Sub-Saharan Africa	0.12 (0.08–0.17)	0.17 (0.11–0.24)	0.33 (0.22–0.48)	0.19 (0.13–0.27)	0.36 (0.32–0.39)
High-income Asia Pacific	0.06 (0.04–0.09)	0.09 (0.06–0.13)	0.02 (0.01–0.03)	0.04 (0.03–0.06)	−2.59(−2.72--2.47)
High-income North America	0.19 (0.11–0.30)	0.17 (0.10–0.26)	0.22 (0.14–0.34)	0.18 (0.11–0.28)	0.18(−0.41–0.77)
North Africa and Middle East	0.15 (0.10–0.22)	0.11 (0.08–0.16)	0.36 (0.24–0.52)	0.14 (0.10–0.20)	0.86 (0.69–1.03)
Oceania	0.00 (0.00–0.01)	0.14 (0.08–0.22)	0.01 (0.00–0.01)	0.12 (0.08–0.19)	−0.79(−0.96--0.62)
South Asia	0.74 (0.45–1.14)	0.17 (0.10–0.26)	1.47 (0.92–2.24)	0.19 (0.12–0.28)	0.29 (0.07–0.50)
Southeast Asia	0.36 (0.24–0.54)	0.18 (0.12–0.27)	0.59 (0.38–0.90)	0.21 (0.14–0.32)	0.40 (0.29–0.52)
Southern Latin America	0.04 (0.02–0.06)	0.20 (0.11–0.32)	0.03 (0.02–0.05)	0.13 (0.08–0.21)	−0.72(−0.98--0.47)
Southern Sub-Saharan Africa	0.07 (0.05–0.10)	0.33 (0.22–0.48)	0.14 (0.09–0.20)	0.41 (0.26–0.60)	0.45(−0.51–1.41)
Tropical Latin America	0.15 (0.09–0.23)	0.23 (0.14–0.35)	0.16 (0.10–0.24)	0.19 (0.11–0.28)	−0.78(−0.92--0.65)
Western Europe	0.36 (0.21–0.57)	0.25 (0.14–0.40)	0.22 (0.14–0.32)	0.17 (0.11–0.25)	−1.75(−1.99--1.51)
Western Sub-Saharan Africa	0.16 (0.10–0.26)	0.23 (0.14–0.36)	0.46 (0.29–0.68)	0.24 (0.15–0.35)	0.18 (0.12–0.24)

ASMR, Age-standardized mortality rate; EAPC, Estimated annual percentage change; CI, Confidence interval; SDI, Socio-demographic index; UI, Uncertainty interval.

**Table 4:** DALYs and ASDR of NAFLD in 1990 and 2021, and temporal trends.

	1990		2021		1990–2021 EAPC of ASDR (95%CI)
	DALYs, No. × 10^3^	ASDR per 100,000 (95% UI)	DALYs, No. × 10^3^	ASDR per 100,000 (95% UI)	
Global	225.36 (153.05–328.28)	10.28 (6.98–14.98)	377.55 (246.02–548.56)	12.69 (8.27–18.44)	0.65 (0.55–0.75)
Socio-demographic index					
Low SDI	19.32 (13.22–27.86)	10.48 (7.17–15.12)	47.23 (31.68–67.56)	10.52 (7.05–15.05)	−0.03(−0.11–0.04)
Low-middle SDI	49.28 (32.17–72.74)	10.87 (7.10–16.04)	102.02 (66.03–148.25)	12.71 (8.23–18.47)	0.52 (0.41–0.63)
Middle SDI	85.76 (59.22–123.47)	11.39 (7.87–16.40)	129.12 (84.85–190.19)	13.92 (9.15–20.51)	0.48 (0.38–0.57)
High-middle SDI	37.75 (25.84–54.28)	8.34 (5.71–11.99)	65.49 (40.62–100.77)	14.87 (9.23–22.89)	2.12 (1.67–2.58)
High SDI	32.99 (20.15–50.85)	9.51 (5.81–14.65)	33.38 (21.26–49.44)	9.45 (6.02–14.00)	−0.29(−0.54--0.04)
Region					
Andean Latin America	4.70 (2.75–7.24)	30.42 (17.78–46.82)	7.16 (4.33–11.07)	26.44 (15.97–40.88)	−0.64(−0.82--0.47)
Australasia	0.48 (0.31–0.71)	5.92 (3.74–8.73)	0.75 (0.52–1.06)	7.20 (5.00–10.13)	0.66 (0.51–0.81)
Caribbean	2.82 (1.67–4.40)	18.97 (11.23–29.59)	3.83 (2.17–6.11)	21.05 (11.90–33.55)	0.40 (0.04–0.76)
Central Asia	4.77 (2.98–7.21)	16.77 (10.46–25.35)	14.03 (8.18–22.27)	37.53 (21.88–59.57)	2.21 (1.79–2.62)
Central Europe	5.32 (3.12–8.35)	11.36 (6.67–17.81)	4.69 (2.69–7.49)	13.39 (7.68–21.40)	0.09(−0.38–0.55)
Central Latin America	18.10 (10.57–28.49)	26.51 (15.49–41.74)	36.65 (21.87–57.29)	36.23 (21.61–56.63)	0.84 (0.61–1.07)
Central Sub-Saharan Africa	2.52 (1.42–4.06)	12.15 (6.84–19.56)	6.34 (3.47–10.05)	11.71 (6.42–18.57)	−0.11(−0.25–0.03)
East Asia	37.47 (28.07–49.72)	6.62 (4.96–8.79)	25.55 (17.95–35.09)	5.33 (3.75–7.33)	−1.30(−1.65--0.95)
Eastern Europe	6.69 (3.99–10.37)	7.80 (4.66–12.09)	40.03 (21.65–65.40)	60.49 (32.72–98.84)	6.86 (5.57–8.16)
Eastern Sub-Saharan Africa	7.24 (5.02–10.25)	10.22 (7.08–14.45)	20.24 (13.61–28.90)	11.55 (7.77–16.50)	0.32 (0.28–0.37)
High-income Asia Pacific	3.36 (2.28–4.91)	4.98 (3.38–7.28)	1.22 (0.82–1.80)	2.41 (1.62–3.55)	−2.58(−2.70--2.45)
High-income North America	10.84 (6.47–16.99)	9.56 (5.71–14.99)	12.77 (8.28–19.34)	10.37 (6.72–15.70)	0.23(−0.34–0.81)
North Africa and Middle East	9.22 (6.25–13.04)	6.89 (4.67–9.74)	21.55 (14.67–30.77)	8.48 (5.77–12.10)	0.82 (0.67–0.98)
Oceania	0.23 (0.13–0.36)	8.59 (4.78–13.56)	0.42 (0.26–0.64)	7.46 (4.61–11.40)	−0.81(−0.98--0.65)
South Asia	43.93 (27.31–67.27)	10.18 (6.33–15.58)	87.30 (55.30–131.28)	11.04 (6.99–16.60)	0.27 (0.05–0.49)
Southeast Asia	21.73 (14.53–32.40)	11.03 (7.38–16.45)	34.72 (22.22–52.02)	12.52 (8.01–18.76)	0.33 (0.22–0.44)
Southern Latin America	2.18 (1.22–3.54)	11.45 (6.39–18.53)	1.97 (1.19–3.06)	7.65 (4.60–11.84)	−0.69(−0.94--0.43)
Southern Sub-Saharan Africa	4.20 (2.85–6.04)	19.43 (13.18–27.94)	8.14 (5.29–11.83)	23.92 (15.54–34.75)	0.41(−0.54–1.38)
Tropical Latin America	8.72 (5.34–13.34)	13.56 (8.30–20.74)	9.49 (5.87–14.07)	10.75 (6.65–15.94)	−0.81(−0.94--0.68)
Western Europe	20.85 (12.14–32.59)	14.47 (8.43–22.61)	12.69 (7.91–18.54)	9.78 (6.10–14.29)	−1.71(−1.93--1.48)
Western Sub-Saharan Africa	9.98 (6.11–15.62)	13.95 (8.54–21.83)	27.99 (17.71–41.08)	14.64 (9.26–21.48)	0.17 (0.11–0.23)

ASDR, Age-standardized DALYs rate; EAPC, Estimated annual percentage change; CI, Confidence interval; SDI, Socio-demographic index; UI, Uncertainty interval.

### NAFLD burden at national level

**Figure 1:**
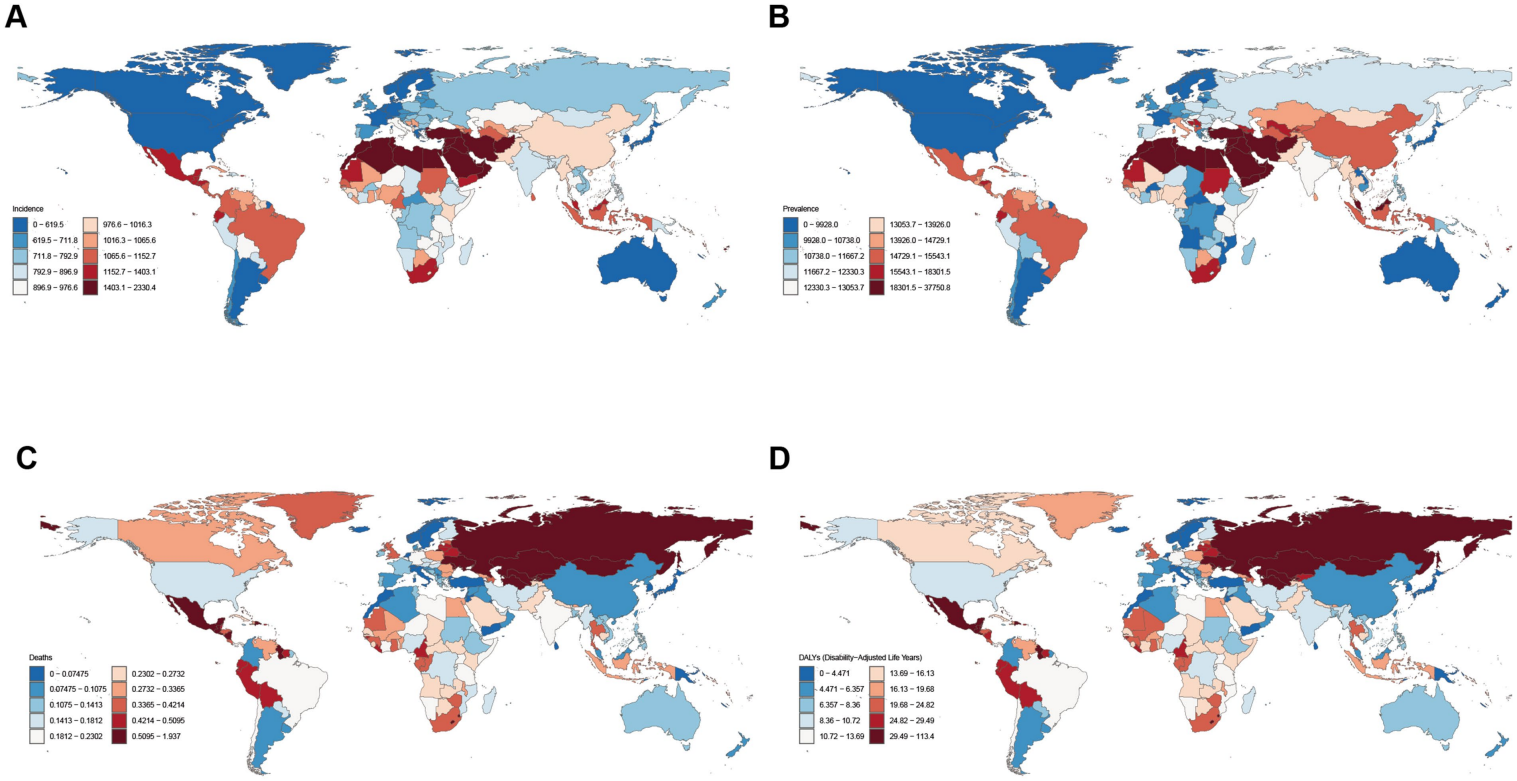
Global disease burden of NAFLD in youths and young adults for both sexes in 204 countries and territories. **(A)** ASIR of NAFLD in 2021; **(B)** ASPR of NAFLD in 2021; **(C)** ASMR of NAFLD in 2021; **(D)** ASDR of NAFLD in 2021.

At the national level in 2021, Egypt exhibited the highest ASIR of NAFLD (2330.40 per 100,000 population; 95% UI: 2041.73–2653.26), for which Jordan (2052.80 per 100,000 population; 95% UI: 1785.58–2342.55) and Iran (2010.53 per 100,000 population; 95% UI: 1748.18–2300.74) followed. Canada had the lowest ASIR of NAFLD (460.66 per 100,000 population; 95% UI: 394.88–538.15), followed by Finland (479.49 per 100,000 population; 95% UI: 410.52–555.29) and Japan (501.20 per 100,000 population; 95% UI: 434.42–577.51). Qatar and Kuwait presented the highest ASPR of NAFLD (37750.82 per 100,000 population, 95% UI: 32195.43–44266.11; 37117.35 per 100,000 population, 95% UI: 31920.34–43371.92). Japan and Finland demonstrated the lowest ASPR of NAFLD (8120.66 per 100,000 population, 95% UI: 7010.11–9476.07; 8196.38 per 100,000 population, 95% UI: 7046.45–9631.22) (Figures 1A,B). In 2021, Turkmenistan and the Russian Federation recorded the highest ASMR of NAFLD (1.94 per 100,000 population, 95% UI: 1.05–3.29; 1.15 per 100,000 population, 95% UI: 0.60–1.85) and the highest ASDR for NAFLD (113.41 per 100,000 population, 95% UI: 61.05–193.69; 64.04 per 100,000 population, 95% UI: 33.68–103.03) (Figures 1C,D).

**Figure 2:**
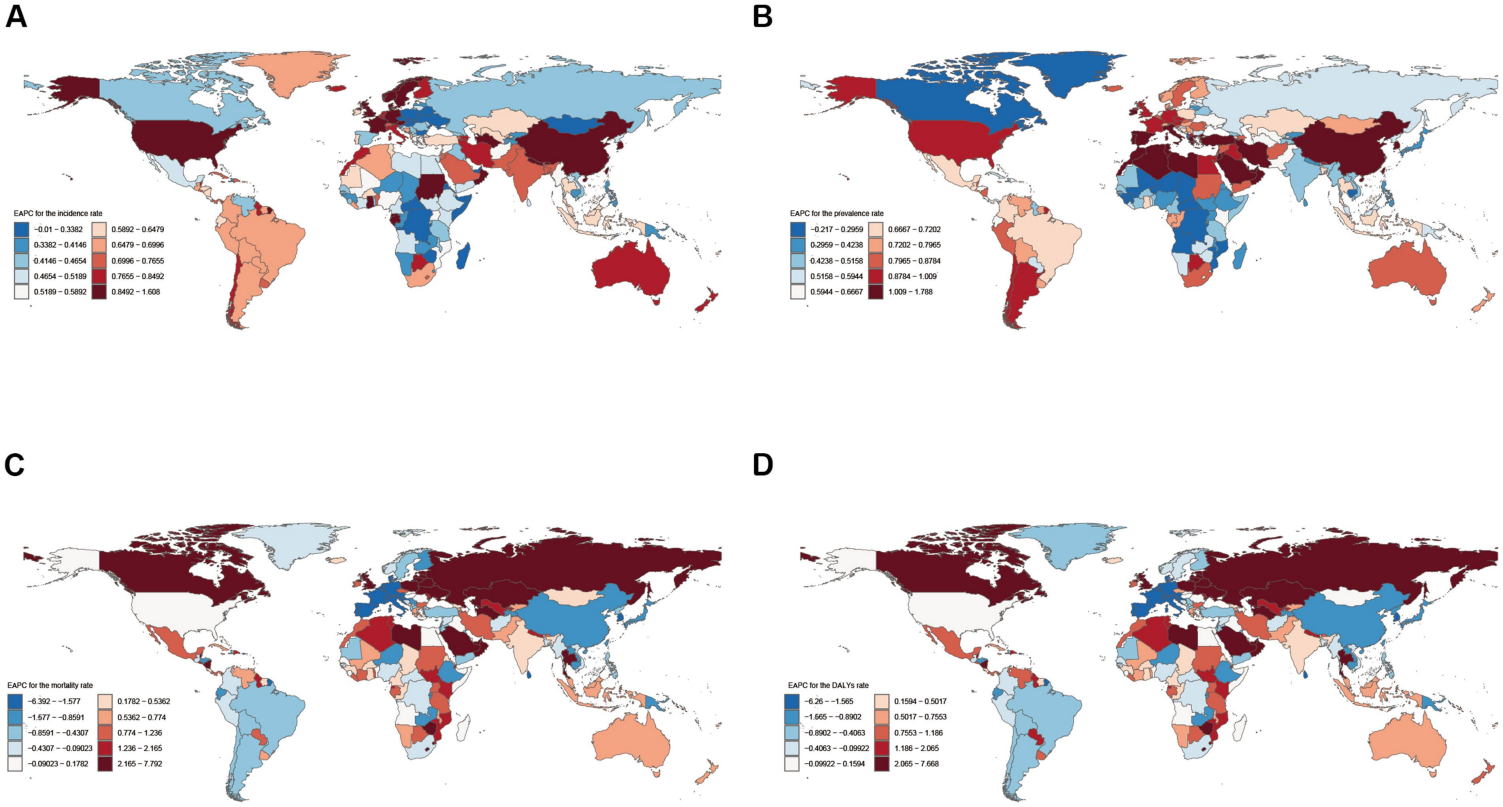
Global temporal trend of NAFLD burden in youths and young adults for both sexes in 204 countries and territories from 1990 to 2021. **(A)** EPAC of the ASIR in NAFLD; **(B)** EPAC of the ASPR in NAFLD; **(C)** EPAC of the ASMR in NAFLD; **(D)** EPAC of the ASDR in NAFLD. EAPC, Estimated annual percentage change.

At the national level from 1990 to 2021, ASIR increased in all countries and territories apart from Japan (EAPC: −0.0099). ASPR also revealed a rise in all countries and areas except for Timor-Leste (EAPC: −0.22) and Northern Mariana Islands (EAPC: −0.15). These estimates suggest that the burden of NAFLD continues to rise among youths and young adults globally. ASIR showed the highest ASIR growth rate in Equatorial Guinea (EAPC: 1.61), followed by Nepal (EAPC: 1.07). Iran registered the highest ASPR growth rate (EAPC: 1.79), followed by Saudi Arabia (EAPC: 1.55) (Figures 2A,B). ASMR and ASDR have increased or decreased in various countries around the globe. Russian Federation had the highest growth rates of ASMR (EAPC: 7.79) and ASDR (EAPC: 7.67) (Figures 2C,D).

### NAFLD burden at regional level

**Figure 3:**
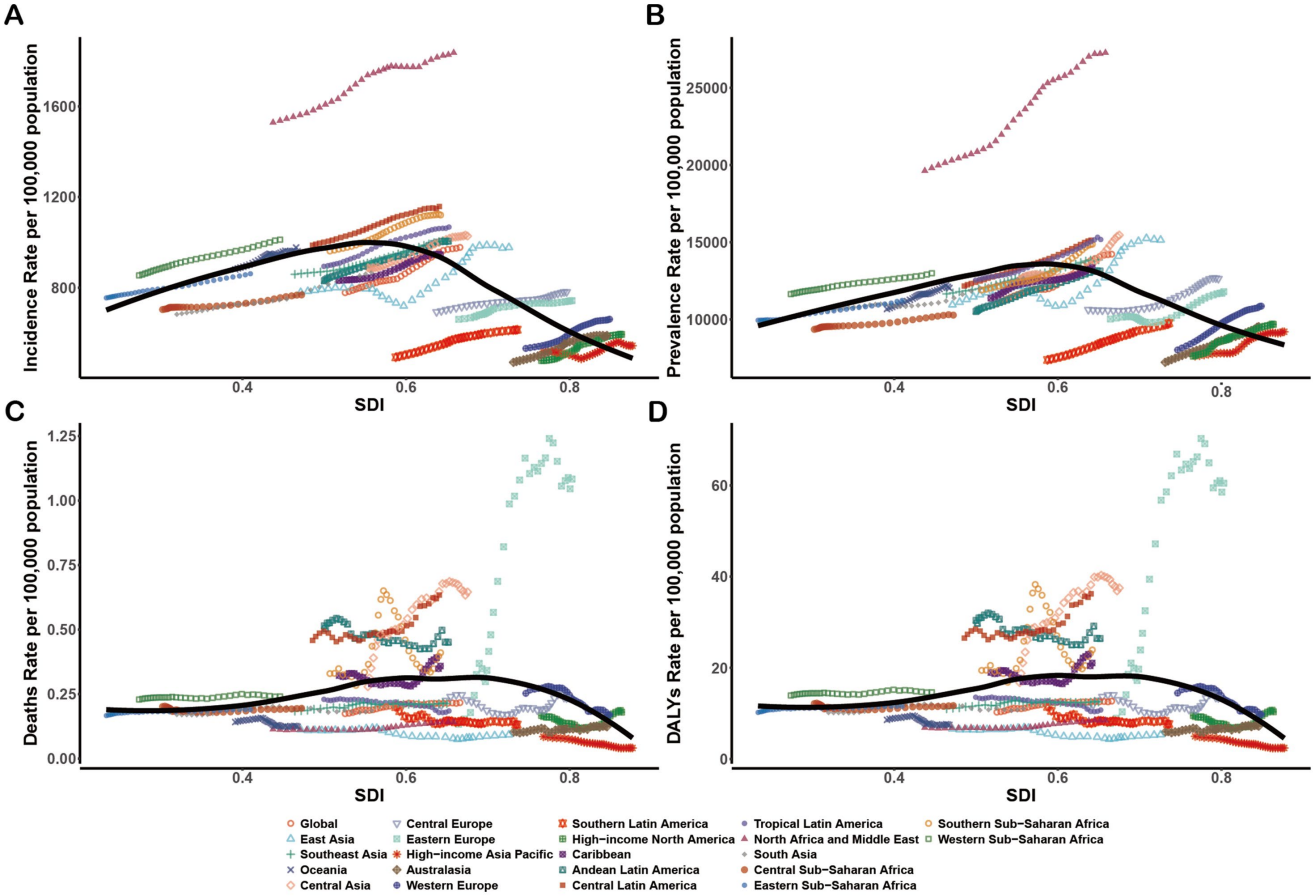
Age-standardized rates of NAFLD in youths and young adults among regions based on SDI from 1990 to 2021. For the black trend line, we fit smooth splines using the Locally Weighted Scatterplot Smoothing method, which automatically determines the degree, number, and location of nodes(knots) on the basis of the data and the span parameter. **(A)** ASIR; **(B)** ASPR; **(C)** ASMR; **(D)** ASDR.

**Figure 4:**
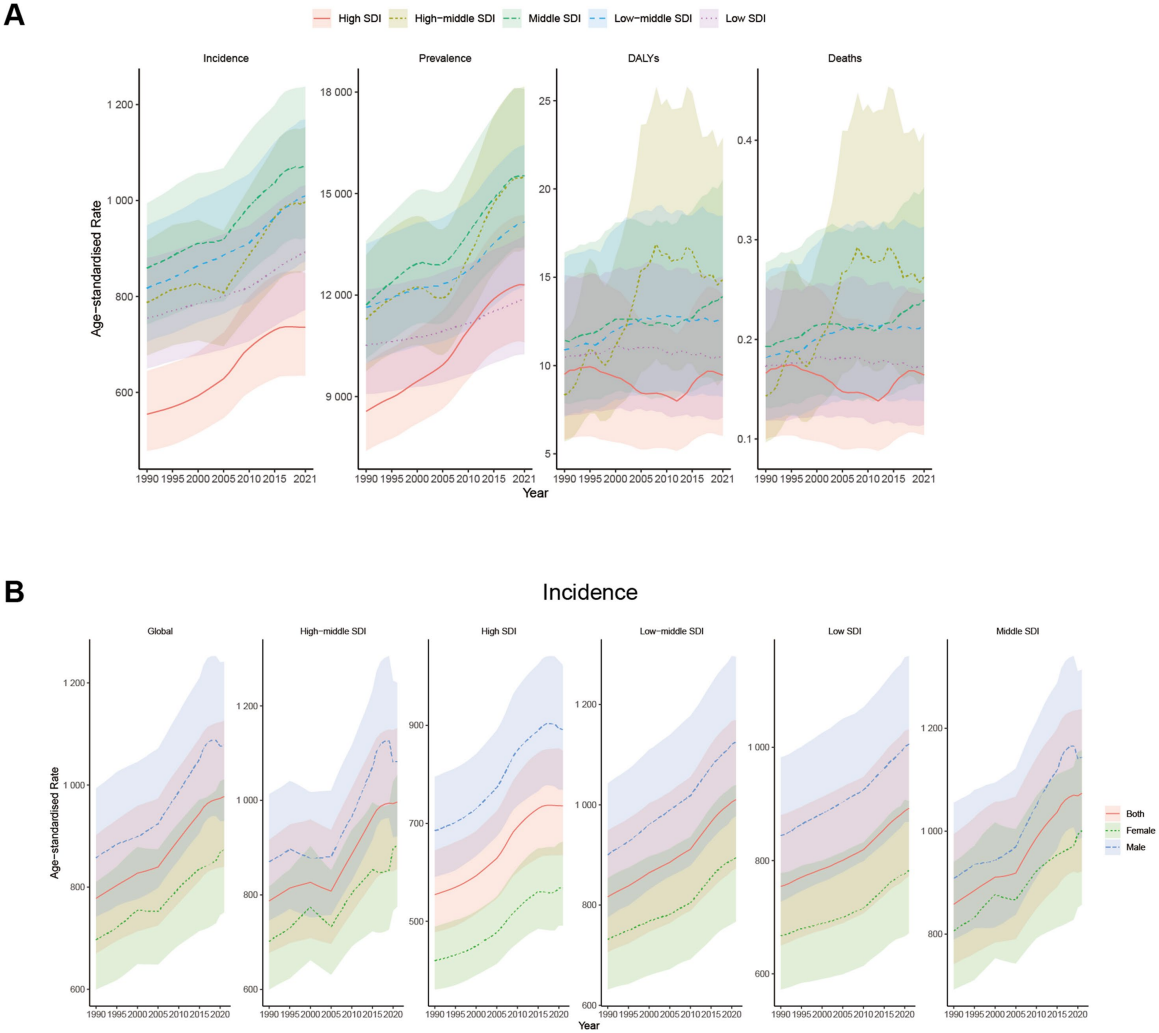
Trends in Global disease burden of NAFLD in youths and young adults at different SDI levels from 1990 to 2021. Shaded areas represent 95% uncertainty intervals (95% UI). **(A)** Trends in ASIR, ASPR, ASMR, and ASDR at different SDI levels. **(B)** Trends in ASIR at different SDI levels with different sexes.

At the regional level, ASIR and ASPR presented an inverted-U shape relationship with SDI (Figures 3A,B). Middle SDI exhibited the highest ASIR and ASPR from 1990 to 2021, whereas High SDI and Low SDI revealed the lowest ASIR and ASPR from 1990 to 2021 (Tables 1, 2; Figure 4A). In 1990, ASMR and ASDR were lowest for high-middle SDI. In contrast, in 2021, ASMR and ASDR were highest for high-middle SDI and lowest for high SDI (Tables 3, 4; Figure 4A).

From 1990 to 2021, all 21 regions demonstrated a sustained upward trend in ASIR and ASPR (Tables 3, 4; Figures 3A,B). ASIR (1836.44 per 100,000 population; 95% UI: 1587.68–2091.66) and ASPR (27269.21 per 100,000 population; 95% UI: 23656.38–31525.15) ranged highest in North Africa and Middle East (Tables 1, 2), exceeding the expected levels from 1990 to 2021 based on the SDI (Figures 3A,B). The ASIR growth rates increased the most in East Asia (EAPC: 0.85) and High-income North America (EAPC: 0.83). North Africa and Middle East had the most significant average annual ASPR increases (EAPC: 1.20), followed by East Asia (EAPC: 1.01) (Tables 1, 2).

In 2021, Eastern Europe had the highest ASMR (1.08 per 100,000 population; 95% UI: 0.57–1.77) and ASDR (60.49 per 100,000 population; 95% UI: 32.72–98.84) (Tables 3, 4), rising above the expected levels based on the SDI from 1990 to 2021 (Figures 3C,D). Meanwhile, Eastern Europe also witnessed the highest ASMR (EAPC: 6.96) and ASDR (EAPC: 6.86) increased rates (Tables 3, 4).

### Age and sex patterns

**Figure 5:**
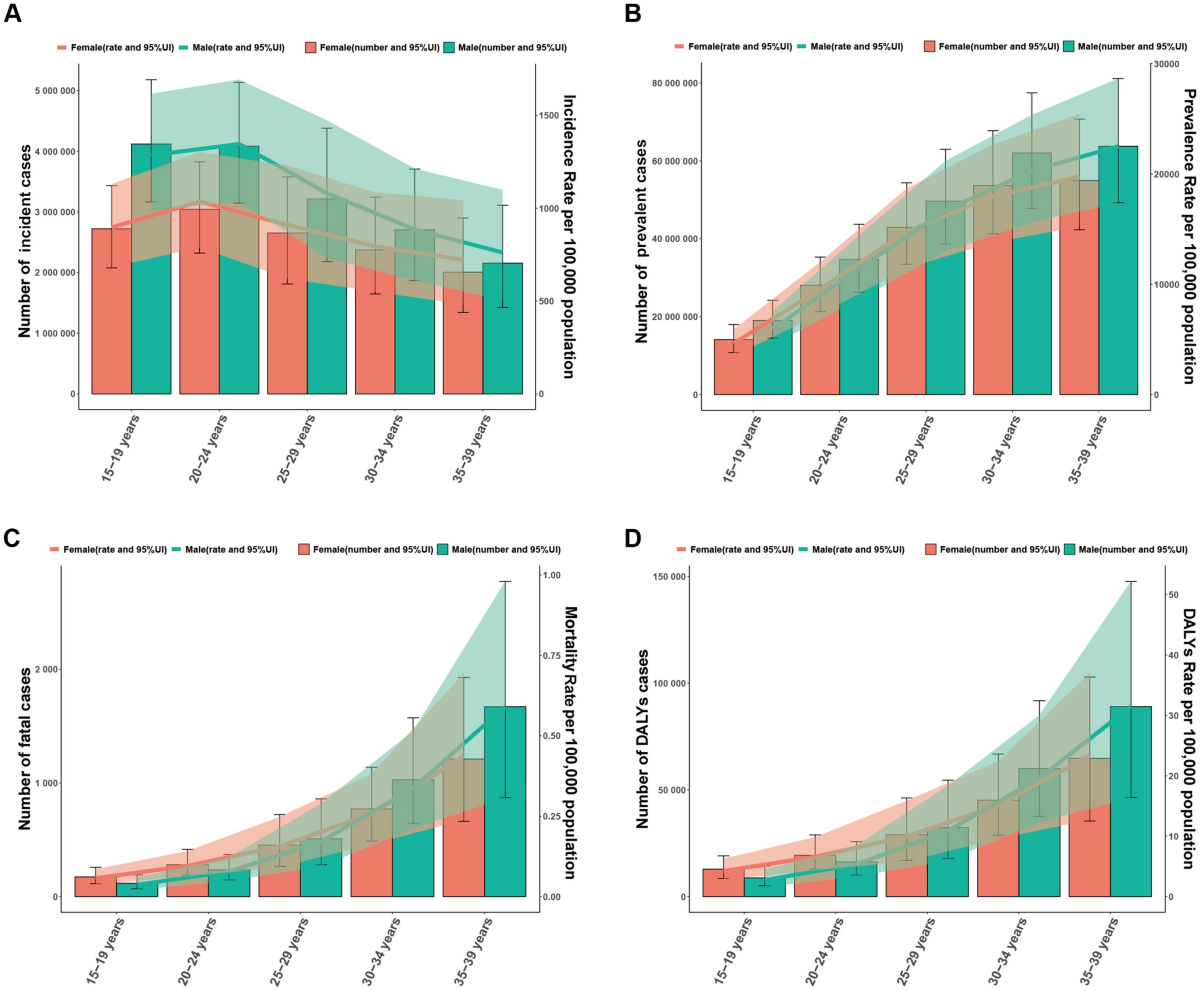
Global disease burden of NAFLD in males and females by different age group in 2021. Shaded areas represent 95% uncertainty intervals (95% UI). The trend line is the line connecting the rates of different age groups. **(A)** Incident cases and ASIR. **(B)** Prevalent cases and ASPR. **(C)** Fatal cases and ASMR. **(D)** DALYs and ASDR.

In 2021, the 20–24 and 15–19 age groups indicated the highest incidence numbers and rates for NAFLD in youths and young adults, which gradually declined with age (Figure 5A). The incidence of NAFLD in the 15–24 years group was higher than other people, reflecting a tendency toward a younger onset of NAFLD. Among youths and young adults aged 15–39 years, the number of incident cases and incident rates of NAFLD were significantly higher in males than in females, which may imply that young males are more susceptible to NAFLD (Figures 4B, 5A). In 2021, the number of prevalent cases and prevalent rates increased progressively with age, reaching a slightly higher number and prevalence in males than in females (Figure 5B).

In 2021, the number of fatal cases and DALYs rose with age. At the same, ASMR and ASDR were observed to exhibit a progressive increase with advancing age. The number of fatal cases, DALYs, and rates in the 15–24 years group were lower in males than in females, while those in the 25–39 years group were higher in males than in females (Figures 5C,D).

**Figure 6:**
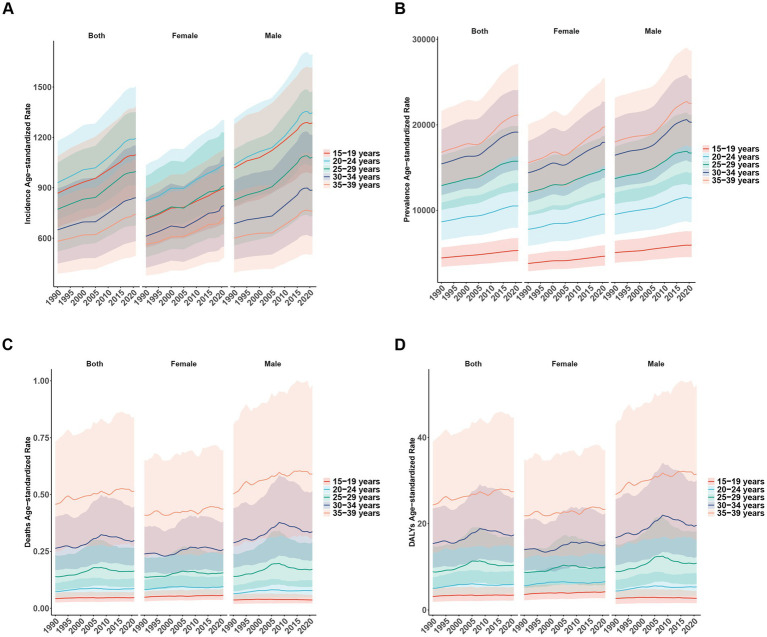
Global disease burden of NAFLD in both sexes and different age groups from 1990 to 2021. Shaded areas represent 95% uncertainty intervals (95% UI). **(A)** ASIR; **(B)** ASPR; **(C)** ASMR; **(D)** ASDR.

From 1990 to 2021, ASIR and ASPR demonstrated a continuous upward trend in both sexes and all various age groups. The 20–24 age group recorded the highest ASIR, while the 35–39 age group registered the highest ASPR (Figures 6A,B). There is no clear pattern of changes in ASMR and ASDR in all age groups (Figures 6C,D).

### Risk factors attributable to NAFLD burden

**Figure 7:**
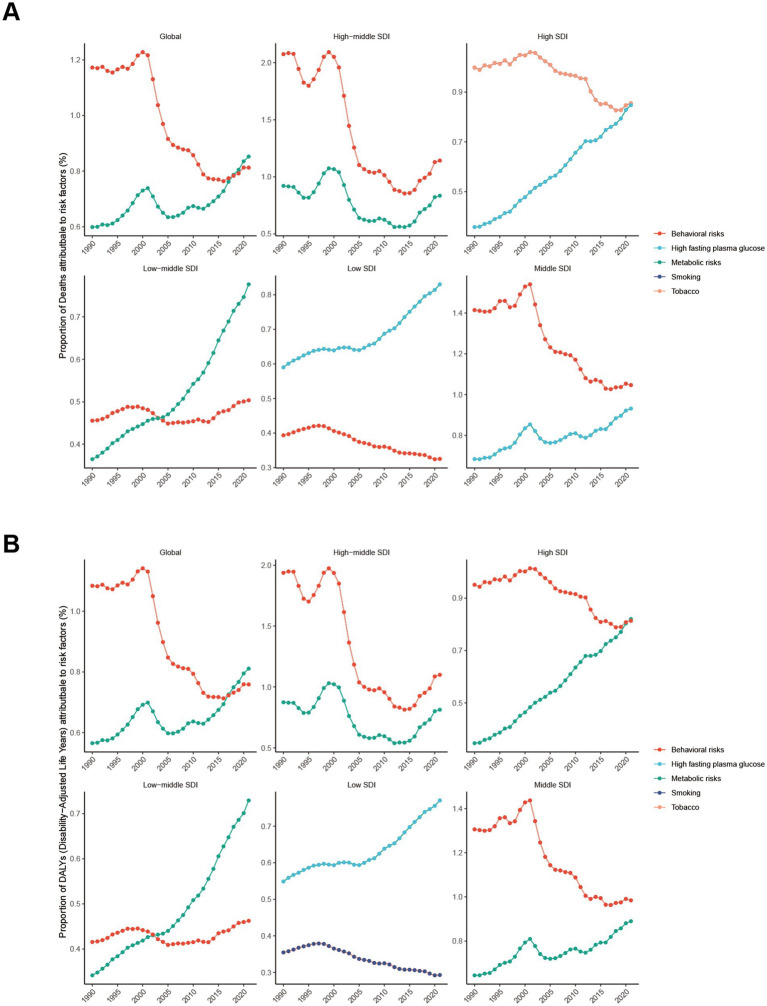
Main risk factors for NAFLD from 1990 to 2021. **(A)** The proportion of deaths attributed to risk factors. **(B)** The proportion of DALYs attributed to risk factors.

To further delineate risk factors for NAFLD, we carried out a detailed analysis of global data from 1990 to 2021. Metabolic risks (high fasting plasma glucose) and Behavioral risks (smoking or tobacco) were the main attributable risk factors for NAFLD (Figure 7).

Globally, the proportion of deaths attributed to metabolic risks (high fasting plasma glucose) was 0.60% in 1990, increasing to 0.85% in 2021. Consistent with the proportion of deaths, the proportion of DALYs resulting from high fasting plasma glucose was 0.58% in 1990, which grew to 0.81% in 2021. High SDI, middle SDI, low-middle SDI, and low SDI regions all demonstrated significant increases of varying degrees. These revealed the importance of fasting plasma glucose management for NAFLD control (Figures 7A,B).

Regarding the other dominant risk factor, the proportion of deaths attributable to Behavioral risks (smoking or tobacco) was 1.28% in 1990 and reduced to 0.81% in 2021. Similar to the proportion of deaths, the proportion of DALYs declined to 0.76% in 2021. These results indicated a decreasing effect of smoking or tobacco on deaths and DALYs from NAFLD in young people (Figures 7A,B).

### Projection of NAFLD

**Figure 8:**
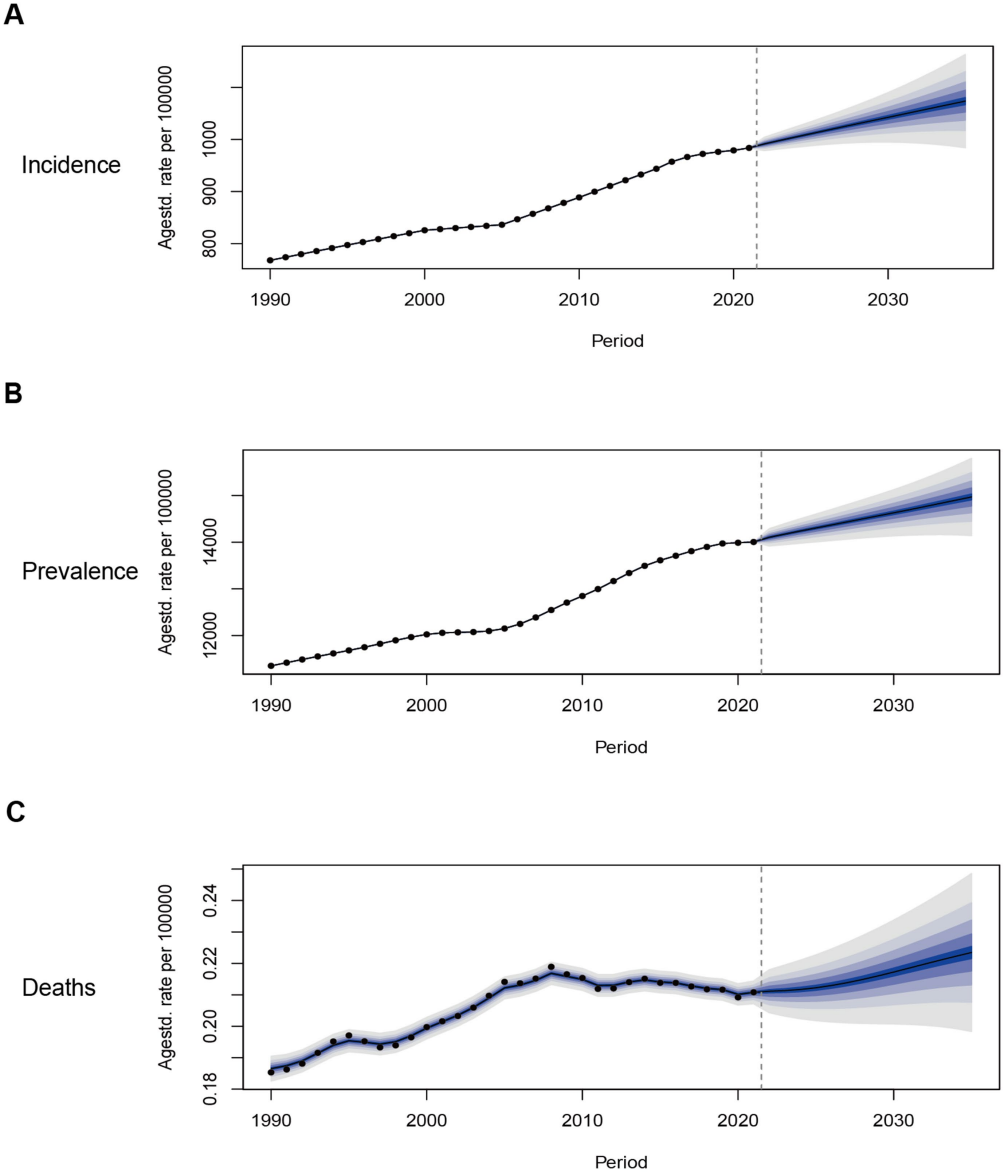
Trends in the NAFLD-related ASIR, ASPR, ASMR in the Global from 1990 to 2035 (BAPC models): observed (dashed lines) and predicted rates (solid lines). The blue region shows the upper and lower limits of the 95% confidence intervals (95% CI). **(A)** Trends in ASIR. **(B)** Trends in ASPR. **(C)** Trends in ASMR.

To learn about the trends of NAFLD in youths and young adults aged 15–39 years after 2021, we predicted ASIR, ASPR, and ASMR from 2021 to 2035 with Bayesian age-period-cohort models (Figure 8). The projections revealed that the ASIR would continue to rise consistently after 2021, from 983.74 per 100,000 (95% CI: 983.38–984.10) in 2021 to 1073.66 per 100,000 (95% CI: 966.92–1180.39) in 2035 (Figure 8A). In line with ASIR, the ASPR will have a continuing increase from 14004.64 per 100,000 (95% CI: 14003.31–14005.98) in 2021 to 14969.49 per 100,000 (95% CI: 13982.54–15956.15) in 2035 (Figure 8B). The overall burden of disease will continually grow over time. ASMR may also increase with time, from 0.211 per 100,000 (95% CI: 0.206–0.215) in 2021 to 0.223 per 100,000 (95% CI: 0.194–0.253) in 2035 (Figure 8C).

## Discussion

For more than three decades, the Global Burden of Disease (GBD) study has systematically and comprehensively recorded and analyzed health data worldwide, stratified by age, sex, and time. The study quantifies the burden of diseases, injuries, and risk factors across different regions and populations, helping policymakers, health experts, and researchers better understand the diversity and trends in global health issues (15, 19). Our study capitalized on the existing literature and provided updated epidemiological insights into the burden of NAFLD in young and young adults with the latest GBD 2021 data (20). We utilized cutting-edge statistical techniques, such as EAPC and ASR, to thoroughly analyze the global temporal trends associated with NAFLD. Furthermore, our study employed BAPC methods to project future trends, providing prospective insights for formulating targeted prevention and control strategies. Specifically, our research centered on the critical age group of 15–39 years, aiming to shed light on the significant burden and far-reaching implications of NAFLD among young people globally. This stage of life marks a pivotal period during which NAFLD can pose undeniable challenges to their quality of life, academic progress, career development, and even social interactions. The high prevalence, incidence, and disability-adjusted life years (DALYs) of NAFLD in this critical age group underscore its considerable impact on the health of this population. Consequently, we urgently appeal to establish age-specific interventions and support systems that can mitigate the long-term detrimental effects of this chronic disease and significantly enhance the quality of life for this group.

The global prevalence of NAFLD has soared dramatically from 1990 to 2021, a worrying trend further reflected in the increasing incidence of NAFLD among young individuals aged 15–39. Both age-standardized incidence rate (ASIR) and age-standardized prevalence rate (ASPR) have increased markedly within this age group. Notably, at the national level, most countries and regions have experienced growth in ASIR and ASPR from 1990 to 2021. The reasons underlying this escalating trend are multifaceted. While urbanization and its consequences, such as unhealthy eating habits and sedentary lifestyles, are frequently credited as culprits, the rising body mass index (BMI) in rural areas contributes significantly to the global obesity trend (21). It implies that the burden of NAFLD continues to increase among young adults globally.

There are complicated relationships between NAFLD burden and SDI, as demonstrated by the results of GBD data analysis. From 1990 to 2021, ASIR and ASPR ranged from the highest for moderate SDI to the lowest for high SDI and low SDI. The prevalence of NAFLD varies in various regions and countries due to differences in economic development, changes in lifestyle, diet structure, medical and health conditions, and many other aspects (21–23). The burden of NAFLD differs across SDI regions and countries, highlighting the importance of implementing targeted interventions and public health strategies.

Our decomposition analysis revealed intriguing trends in several regions, including Eastern Europe, East Asia, North Africa, the Middle East. In 2021, the ASIR and ASPR of the 15–39 age group in North Africa and the Middle East were the highest, with the fastest growing ASIR in East Asia and high-income North America. These findings are indeed unsurprising, as they profoundly insight into the congruence between the prevalence of NAFLD and the spreading trajectories of obesity and T2DM, two major global health crises that are particularly severe in the Middle East and North Africa regions (3, 24). At the same time, variations in access to healthcare and the level of medical care remain the root causes for the enormous heterogeneity in prevalence and incidence rates across countries (25). Furthermore, in East Asia and high-income North America, alterations in lifestyle factors such as diet, stress levels, and sleep patterns may also account for the prevalence and severity of NAFLD (26). Meanwhile, the rising prevalence of T2DM in high-income North America also underlies the increase in NAFLD prevalence, potentially owing to environmental factors, psychosocial factors, and lifestyle modifications. It is worth exploring further that Eastern Europe had the highest ASMR and ASDR in 2021, possibly resulting from economic, dietary, and medical disparities.

Our research indicated that the incidence of NAFLD was higher in the 15–24 age group than in other age groups, mirroring the trend of younger NAFLD onset. Given that the pathogenesis of NAFLD is related to fat accumulation in hepatocytes and the growing obesity rate among young people, this provides a plausible explanation for why the number of young patients is increasing (27, 28). Alarmingly, more people are suffering from NAFLD at an earlier age, which implies that they have more time to develop severe complications (29). In 2021, the number of deaths and DALYs increased with age, likely because NAFLD is a chronic disease that advances to NASH and even liver cancer in later stages, leading to death (30). Meanwhile, the incidence rate of NAFLD among male youths and young adults aged 15–39 is significantly higher than that of females. It is consistent with previous findings that estrogen prevents NAFLD (31).

In our study, high fasting plasma glucose was one of the major attributable risk factors for NAFLD in the GBD database. Numerous studies have unanimously demonstrated that hyperglycemia can carry an elevated risk of NAFLD (32, 33). Patients with hyperglycemia often exhibit insulin resistance and metabolic syndrome, both of which increase the incidence and accelerate the development of NAFLD (34, 35). Another risk factor is smoking. Our research indicated a decreasing effect on NAFLD-related deaths and disability-adjusted life years (DALYs) attributable to smoking among young people. Over the past three decades, the global smoking rate has declined by 27.5% (36). Parallel to this, in 2007, the World Health Organization approved a practical and cost-effective plan to curb the tobacco epidemic and protect people from its harmful effects (37).

To gain a profound insight into the trends in NAFLD among youth and young adults aged 15–39 years after 2021, we applied a Bayesian age-period-cohort model to accurately predict and analyze the ASIR, ASPR, and ASMR within this demographic cohort from 2021 to 2035. The global trend of NAFLD will be a continued increase in prevalence and a growing disease burden over time. It is attributable to various factors such as lifestyle alterations, the prevalence of metabolic diseases, uneven development of medical resources, and genetic factors. Comprehensive prevention and control measures are essential to effectively address the global challenge of NAFLD (38, 39).

This study, nonetheless, inherently has several notable limitations. Firstly, despite employing rigorous statistical methods in our research, disparities in health information systems and reporting mechanisms in diverse countries and regions (especially in low-and middle-income countries and conflict zones) may lead to incomplete and biased data. These factors, in turn, may affect the accuracy of our findings. Furthermore, the lack of data on other relevant parameters prevented us from identifying other potential interrelated factors associated with NAFLD, necessitating further investigation. Lastly, we did not conduct subgroup analyses based on whether NAFLD patients had comorbid metabolic syndrome, were undergoing pharmacological treatment, or had other factors.

## Conclusion

From 1990 to 2021, the global burden of NAFLD in people aged 15–39 years has increased significantly, revealing marked variations between SDI regions, countries, age groups, and genders. We utilized the BAPC model to describe trends in NAFLD in terms of age, period, and cohort, from which we projected future shifts to predict the disease burden in NAFLD. The results of the BAPC model indicated a continued increase in the global prevalence of NAFLD and a sustained rise in the disease burden. By 2035, NAFLD is anticipated to place a significant strain on society. Therefore, future research should prioritize identifying risk factors, enhance diagnostic and treatment options, and develop effective prevention strategies to reduce the global burden of NAFLD in adolescents and young adults.

## Data Availability

The datasets for this article are available from the Global Health Data Exchange query tool (http://ghdx.healthdata.org/gbd-results-tool). All data will be made available on request to the corresponding author.

## Footnotes

^1^ http://ghdx.healthdata.org/gbd-results-tool
